# Nickel pyrithione induces apoptosis in chronic myeloid leukemia cells resistant to imatinib via both Bcr/Abl-dependent and Bcr/Abl-independent mechanisms

**DOI:** 10.1186/s13045-016-0359-x

**Published:** 2016-11-25

**Authors:** Xiaoying Lan, Chong Zhao, Xin Chen, Peiquan Zhang, Dan Zang, Jinjie Wu, Jinghong Chen, Huidan Long, Li Yang, Hongbiao Huang, Bing Z. Carter, Xuejun Wang, Xianping Shi, Jinbao Liu

**Affiliations:** 1 Department of Pathophysiology, State Key Lab of Respiratory Disease, Protein Modification and Degradation Laboratory, Guangzhou Medical University, Guangzhou, Guangdong 511436 China; 2 Department of Leukemia, Section of Molecular Hematology and Therapy, The University of Texas M.D. Anderson Cancer Center, Houston, TX 77030 USA; 3 Division of Basic Biomedical Sciences, Sanford School of Medicine of the University of South Dakota, Vermillion, SD 57069 USA; 4 Department of Pathophysiology, Protein modification and Degradation Laboratory, Guangzhou Medical University, Guangzhou, Guangdong 510182 People’s Republic of China

**Keywords:** Nickel pyrithione, Apoptosis, Chronic myelogenous leukemia, Imatinib resistance, Bcr-Abl

## Abstract

**Background:**

Acquired imatinib (IM) resistance is frequently characterized by Bcr-Abl mutations that affect IM binding and kinase inhibition in patients with chronic myelogenous leukemia (CML). Bcr-Abl-T315I mutation is the predominant mechanism of the acquired resistance to IM. Therefore, it is urgent to search for additional approaches and targeting strategies to overcome IM resistance. We recently reported that nickel pyrithione (NiPT) potently inhibits the ubiquitin proteasome system via targeting the 19S proteasome-associated deubiquitinases (UCHL5 and USP14), without effecting on the 20S proteasome. In this present study, we investigated the effect of NiPT, a novel proteasomal deubiquitinase inhibitor, on cell survival or apoptosis in CML cells bearing Bcr-Abl-T315I or wild-type Bcr-Abl.

**Methods:**

Cell viability was examined by MTS assay and trypan blue exclusion staining assay in KBM5, KBM5R, K562, BaF3-p210-WT, BaF3-p210-T315I cells, and CML patients’ bone marrow samples treated with NiPT. Cell apoptosis in CML cells was detected with Annexin V-FITC/PI and rhodamine-123 staining followed by fluorescence microscopy and flow cytometry and with western blot analyses for apoptosis-associated proteins. Expression levels of Bcr-Abl in CML cells were analyzed by using western blotting and real-time PCR. The 20S proteasome peptidase activity was measured using specific fluorogenic substrate. Active-site-directed labeling of proteasomal DUBs, as well as the phosphorylation of USP14 was used for evaluating the inhibition of the DUBs activity by NiPT. Mouse xenograft models of KBM5 and KBM5R cells were analyzed, and Bcr-Abl-related proteins and protein biomarkers related to proliferation, differentiation, and adhesion in tumor tissues were detected by western blots and/or immunohistological analyses.

**Results:**

NiPT induced apoptosis in CML cells and inhibited the growth of IM-resistant Bcr-Abl-T315I xenografts in nude mice. Mechanistically, NiPT induced decreases in Bcr-Abl proteins, which were associated with downregulation of Bcr-Abl transcription and with the cleavage of Bcr-Abl protein by activated caspases. NiPT-induced ubiquitin proteasome system inhibition induced caspase activation in both IM-resistant and IM-sensitive CML cells, and the caspase activation was required for NiPT-induced Bcr-Abl downregulation and apoptotic cell death.

**Conclusions:**

These findings support that NiPT can overcome IM resistance through both Bcr-Abl-dependent and Bcr-Abl-independent mechanisms, providing potentially a new option for CML treatment.

## Background

The constitutive activation of the Bcr-Abl tyrosine kinase resulting from the *t*(9;22) chromosomal translocation is necessary for the transformed phenotype of chronic myelogenous leukemia (CML) [1–3]. The Bcr-Abl fusion oncoprotein constitutively activates mitogenic signaling pathways such as MAPK/ERK cascade, PI3K/Akt/mTOR, and STATs pathways [4–6]. The activation of these pathways in Bcr-Abl-expressing cells results in increased expression of several anti-apoptotic proteins (such as Bcl-2, Mcl-1 and XIAP), thereby conferring cell survival advantage [7–9]. Thus, these abnormalities should be targeted when designing novel strategies for the treatment of CML. Imatinib (IM; Gleevec) was developed to selectively inhibit the abnormal tyrosine kinase activity of Bcr-Abl and showed significant efficacy in treatment for CML for inducing cytogenetic and molecular remission [10–12]. However, despite its impressive efficacy, for a portion of patients, IM single-agent therapy is not sufficient to control this disease. Some patients may respond suboptimally, and others fail to respond at all [13, 14]. Among the mechanisms proposed so far to account for the IM resistance, amplification and mutation of Bcr-Abl are believed to be the predominant ones. T315I mutation, the most stubborn point mutation, accounts for about 20% of mutations within the Abl kinase domain [15–17]. To overcome this resistance, second-generation Abl kinase inhibitors such as nilotinib, dasatinib, and bosutinib have been developed and are effective against a range of Bcr-Abl mutations except T315I [18–20]. Ponatinib, a third-generation TKI, was specifically designed to inhibit BCR-ABL-positive CML cells containing the T315I mutation. Although the initial response to ponatinib is promising in CML patients with single mutations in BCR-ABL, the response in advanced patients is limited because successive use of TKIs leads to the evolution of compounded BCR-ABL kinase domain mutations that show resistance even to ponatinib [21]. Hence, additional strategies to overcome the IM resistance are warranted.

Studies suggest that in addition to post-translational modifications (mainly phosphorylation), abnormalities in Bcr-Abl protein translation and degradation also play critical roles in initiation, development, and induction of drug resistance in CML [10]. Recent data suggest that inhibiting the expression of Bcr-Abl may be a promising strategy [22]. We and others have reported that metal-containing compounds can induce cytotoxicity in cancer cells via targeting the ubiquitin proteasome system (UPS) [23–25]. Pyrithione (PT) possesses well-defined metal-chelating properties, and the zinc complex of pyrithione (ZnPT) was found to exert significant anti-cancer effects [26]. And only recently we have definitely confirmed that nickel pyrithione (NiPT) inhibits the 19S proteasome-associated deubiquitinases (DUBs) USP14 and UCHL5, but not the 20S proteasome peptidases, and the inhibition of proteasome-associated DUBs induces NiPT-mediated cytotoxicity, revealing a novel mechanism for the anti-cancer effects of nickel-containing compounds [27]. Although proteasome inhibitors such as bortezomib (PS-341) and gambogic acid (GA) have been reported to downregulate or inhibit Bcr-Abl expression and induce cell apoptosis in CML cells [28, 29], the role of DUB inhibitors, especially inhibitors of the 19S proteasome-associated DUBs, in Bcr-Abl hematopoietic malignancies remains unknown.

Here, we investigated the anti-neoplastic effects of NiPT on Bcr-Abl wild-type and Bcr-Abl-T315I mutant cell lines, primary cells from CML patients, and mouse IM-resistant xenograft models. The results demonstrate that NiPT can efficiently overcome IM resistance through both Bcr-Abl-dependent and Bcr-Abl-independent mechanisms.

## Methods

### Chemicals

NiPT was synthesized in our laboratory, and a 20-mM stock solution in dimethyl sulfoxide (DMSO) was stored at −20 °C. b-AP15, Suc-LLVY-AMC, and HA-ubiquitin-vinyl sulfone (HA-Ub-VS) were obtained from Boston Biochem (MA, USA). Annexin V, propidium iodide (PI), and rhodamine-123 were purchased from Sigma-Aldrich (St. Louis, MO, USA). Proteasome inhibitor bortezomib (PS-341) and caspase inhibitor z-VAD-fmk were obtained from BD Biosciences (San Jose, CA, USA). Antibodies against c-Abl (C-19), Mcl-1 (S-19), ubiquitin (P4D1), caspase-3, -8, -9, apoptosis-inducing factor (AIF), Bcl-2, USP14, and UCHL5 were from Santa Cruz Biotechnology (Santa Cruz, CA, USA). Antibody against poly(ADP)-ribose polymerase (PARP; clone 4C10-5) was from BD Biosciences. Antibodies against phospho-c-Abl at Y245, phospho-Erk1/2 (T202/Y204), Erk1/2, phospho-CrkL at Y207, CrkL, phospho-Akt, Akt, RNA polymerase II (pol II), phospho-RNA Pol II at Ser2 and Ser5, cyclin-dependent kinase 7 (CDK7), CDK9, IκB-α, p27, phospho-eIF2α, eIF2α, CHOP, HA, and XIAP were from Cell Signaling Technology (Beverly, MA, USA). Antibodies against phospho-STAT5A/B (Y694/Y699; clone 8-5-2), STAT5, Ki67, CD11b/c, and CXCR4 were from Upstate Technology; mouse monoclonal antibody against actin was from Sigma-Aldrich. Antibodies against phospho-USP14 (S241) was from ABGENT. Enhanced chemiluminescence (ECL) reagents were purchased from Amersham Biosciences (Piscataway, NJ, USA).

### Cell culture

The KBM5 cell line expressing the 210 kD BCR-ABL protein and lacking normal c-ABL was derived from a female myeloid CML patient in blast crisis as described previously [30]. KBM5-T315I (KBM5R) cell line, an imatinib-resistant KBM5 subline, was derived from KBM5 by exposing to increasing concentrations of imatinib, leading to the selection of survival clone harboring T315I mutation as previously described [31, 32]. KBM5 cells were cultured in Iscove’s modified Dulbecco’s medium (Gibco-BRL, Gaithersburg, MD) supplemented with 10% fetal bovine serum in 5% CO_2_ at 37 °C. KBM5-T315I cells were routinely maintained in the same medium in the presence of 1 μM imatinib. In the study where KBM5-T315I cells were compared with the parental KBM5 cells, imatinib was washed off and KBM5-T315I cells were cultured in drug-free medium for several days before the experiments. BaF3-Bcr/Ablp210-wt cells and BaF3-Bcr/Abl-T315I cells (kindly provided by Dr. BZ. Carter, University of Texas M. D. Anderson Cancer Center, Houston, USA) were derived from the murine BaF3 cells by transfection using vectors MSCV-BCR/ABL-PAC and MSCV-BCR/ABL-T315I-PAC [31]. Cells were selected in 2 μg/mL puromycin to achieve stable expression as described previously [31]. Upon receipt of KBM5, KBM5R, BaF3-Bcr/Abl-wt, and BaF3-Bcr/Abl-T315I cell lines, cells were expanded and frozen; cells were not passaged for more than 6 months after resuscitation. K562 cell line was obtained from the ATCC and routinely authenticated by karyotyping, short tandem repeat profiling, assessment of cell morphology, and species verification by isoenzymology. BaF3-Bcr/Abl-wt, BaF3-Bcr/Abl-T315I, and K562 cells were cultured in RPMI 1640 medium with 10% fetal bovine serum as described previously [30–33].

Bone marrow samples from CML patients were obtained from the Department of Hematology, the First Affiliated Hospital of Sun Yat-sen University. The use of these materials is approved by the Institutions with the permission of the patients. Totally, 12 patients with CML were recruited in this preclinical study. Mononuclear cells were isolated using Histopaque gradient centrifugation (density 1.077; Pharmacia, Uppsala, Sweden). Contaminating red cells were lysed in 0.8% ammonium chloride solution for 10 min. After isolation, cells were washed with phosphate-buffered saline and suspended in fresh RPMI 1640 culture medium with 15% fetal calf serum as described previously [30]. All drug treatments were not started until the cells were precultured in fresh medium for 24 h.

### Western blot analysis

Whole cell lysates were prepared in RIPA buffer [29] (1× PBS, 1% NP-40, 0.5% sodium deoxycholate, 0.1% SDS) and was supplemented with freshly added 10 mM β-glycerophosphate, 1 mM sodium orthovanadate, 10 mM NaF, 1 mM phenylmethylsulfonyl fluoride, and 1× Roche Complete Mini Protease Inhibitor Cocktail (Roche, Indianapolis, IN). To detect the level of cytochrome C and AIF in the cytosol, the cytosolic fraction was prepared with digitonin extraction buffer (10 mM PIPES [pH 6.8], 0.015% [wt/vol] digitonin, 300 mM sucrose, 100 mM NaCl, 3 mM MgCl_2_, 5 mM EDTA, and 1 mM PMSF) as described previously [29]. Western blot analysis was performed as we previously described, using specific primary antibodies as indicated and appropriate horseradish peroxidase (HRP)-conjugated secondary antibodies as indicated.

### Cell viability assay

MTS assay (CellTiter 96 Aqueous One Solution reagent; Promega, Shanghai, China) was performed to test cell viability. Briefly, 2 × 10^5^/ml cells in 100 μl were treated with NiPT for 48 h. At 3–4 h before culture termination, 20 μl of MTS reagent was added to the wells. The absorbance density was read on a 96-well plate reader (Varioskan Flash 3001, Thermo, Waltham, MA, USA) at wavelength 490 nm. IC_50_ values were calculated as the concentration of the drug required to obtain 50% of maximal inhibition in cell viability.

### Cell death assay

CML cells were treated with increasing concentrations of NiPT for indicated time periods, and 0.4% trypan blue was added to monitor temporal changes in the incidence of cell death under the light microscope. Cell apoptosis was determined by flow cytometry or recorded under an inverted fluorescence microscope using Annexin V-fluoroisothiocyanate (FITC)/PI double staining [29, 30]. Cells were incubated with indicated concentration of NiPT, collected and washed with binding buffer (BD Biosciences Pharmingen), then incubated in working solution (100 μL binding buffer with 0.3 μL Annexin V-FITC) for 15 min in dark. Cells were washed and resuspended with binding buffer. PI (Sigma-Aldrich) was added just before flow cytometric analysis. At the indicated time points, the PI/Annexin V positive cells in the culture dish were imaged with an inverted fluorescence microscope equipped with a digital camera (AxioObsever Z1, Zeiss, Germany).

### Measurement of mitochondrial membrane integrity

The mitochondrial membrane potential (ΔΨm) of NiPT-treated and NiPT-untreated cells were determined by rhodamine-123 (Sigma-Aldrich, St. Louis, MO) staining. Cells were treated with NiPT for 24 h and stained with 1 μM of rhodamine-123 for 1 h at 37 °C. Following the staining, the cells were washed with PBS twice, and then harvested for either imaging with an inverted fluorescence microscope or flow cytometry analysis.

### Peptidase activity assay

Fluorogenic substrate Suc-LLVY-AMC was used to assess chymotrypsin-like activity of the 20S proteasome. To evaluate in vitro proteasome inhibition, leukemia cells were lysed in ice-cold lysis buffer (25 mM Tris-HCl) for 10 min. Equal amounts of protein from each sample were then treated with NiPT or bortezomib for 30 min, and then incubated at 37 °C with 25 nM fluorogenic substrate for 1–2 h. Fluorescence intensity was measured using a spectrophotometer at excitation of 350 nm and emission of 438 nm (Varioskan Flash 3001, Thermo).

### DUB active-site-directed labeling assays

Crude cell lysates extracted from cultured CML cells using the DUB assay buffer (25 mM Tris-HCl pH 7.4, 5 mM MgCl_2_, 20 mM NaCl) were incubated with HA-UbVS for 30 min at 37 °C, followed by boiling in the reducing sample buffer and fractionated via SDS-PAGE. HA-UbVS-labeled DUBs were immunodetected using anti-HA antibodies.

### RNA isolation and reverse transcriptase real-time quantitative polymerase chain reaction (RT^2^-PCR)

Total RNA was extracted from 1 × 10^7^ cells by use of Trizol reagent (Invitrogen). After quantification by spectrophotometry, the first-strand cDNA was synthesized from 500 ng total RNA using the RNA PCR Kit (AMV) Ver.3.0 (TaKaRa, Dalian, China) and random primers. Then 50 ng of total cDNA was used for real-time PCR with the SYBR Premix Ex TaqIIKit (TaKaRa). The PCR was performed using the Applied Biosystems™ 7500 Real-Time PCR Systems. The relative gene expression was analyzed by the Comparative Ct method using 18S ribosomal RNA as the endogenous control. The primers for real-time PCR are as follows: Bcr-Abl forward, 5′- AAG CGC AAC AAG CCC ACT GTC TAT-3′; reverse, 5′-CTT CGT CTG AGA TAC TGG ATT CCT-3′. 18S forward, 5′-AAA CGG CTA CCA CAT CCA AG-3′; reverse, 5′-CCT CCA ATG GAT CCT CGT TA-3′.

### Nude mouse xenograft model

Nude Balb/c mice were bred at the animal facility of Guangzhou Medical University. The mice were housed in a barrier facility with a 12-h light dark cycle, with food and water available ad libitum. 3 × 10^7^ of KBM5 or KBM5R cells were inoculated subcutaneously on the flanks of 5-week-old male nude mice. At 72 h after the inoculation, mice were treated with either vehicle (10% DMSO, 30% polyethylene glycol, and 60% physiological saline, i.p.) or NiPT (2.5 mg/kg of body weight, i.p.) every other day for a total of 11 or 14 days. Tumors were measured every day with the use of calipers. Tumor volumes were calculated by the following formula: *a* ^2^ × *b* × 0.4, where *a* is the smallest diameter and *b* is the diameter perpendicular to *a*. The animals were then euthanized, and tumor xenografts were immediately removed, weighed, and frozen or fixed for biochemical or histological analyses, respectively. All animal studies were conducted with the approval of the Guangzhou Medical University Institutional Animal Care and Use Committee.

### Immunohistochemical staining

Formalin-fixed xenografts were processed, paraffin-embedded, and sectioned with standard techniques. Tumor xenograft sections (4 μm) were immunostained for c-Abl, Ubs, p27, CD11b/c, CXCR4, and Ki67. MaxVisionTM reagent was applied to each slide (MaixinBiol) according to the manufacturer’s instructions. Color was developed with 0.05% diaminobenzidine and 0.03% H_2_O_2_ in 50 mmol/L Tris-HCl (pH 7.6), and the slides were counterstained with hematoxylin. A negative control for every antibody was also included for each xenograft specimen by substituting the primary antibody with preimmune rabbit serum.

### Statistical analysis

All experiments were performed at least thrice, and the results were expressed as mean ± SD where applicable. GraphPad Prism 4.0 software (GraphPad Software) was used for statistical analysis. Student’s *t* test was used to compare the differences between variables. *P* value of <0.05 was considered statistically significant.

## Results

### NiPT decreases viability of both Bcr-Abl wild-type and Bcr-Abl-T315I cells

**Fig. 1:**
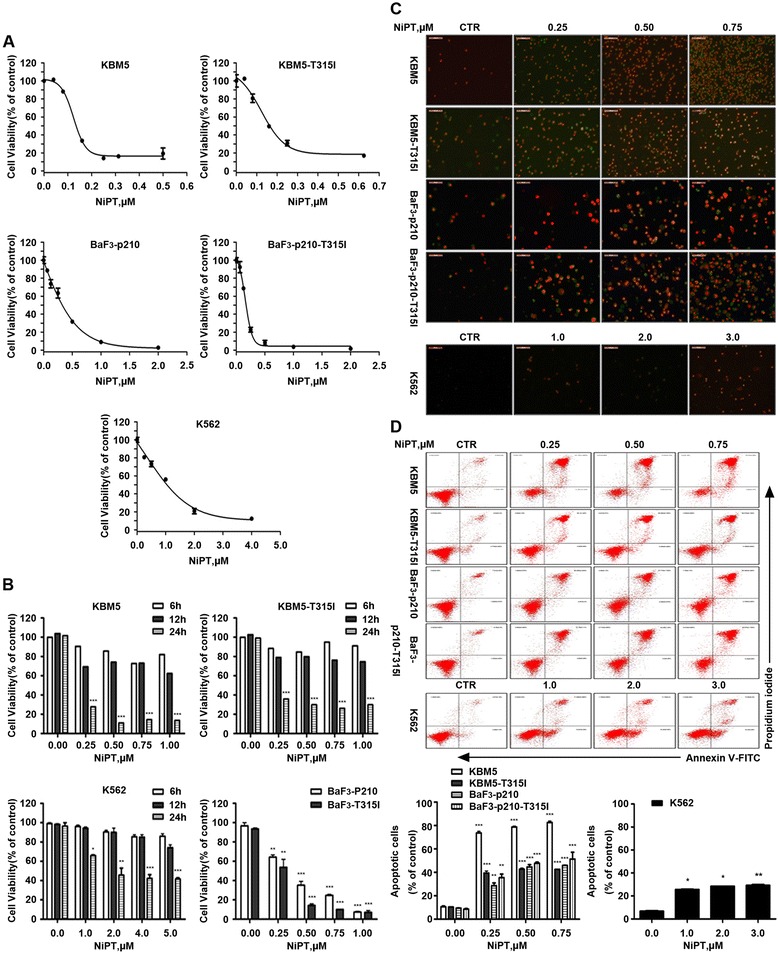
NiPT inhibits cell viability in CML cell lines. **a**, **b** NiPT decreases the viability of both IM-sensitive and IM-resistant CML cell lines. KBM5, KBM5R, K562, BaF3-p210-WT, and BaF3-p210-T315I cells were exposed to NiPT in various concentrations for 48 h, and then were subject to MTS assay. Cell viability was also examined by trypan blue exclusion staining assay. All CML cells were exposed to NiPT followed by trypan blue staining. *Graphs* represent data from three repeats. Mean ± SD (*n* = 3). *P < 0.05,**P < 0.01, ***P < 0.001, versus control group. **c**, **d** NiPT triggers cell apoptosis in CML cells. Cells were treated with different doses of NiPT for 24 h, then propidium iodide (PI) and Annexin V-FITC were added to the culture medium and cell apoptosis were observed by recording the Annexin V-FITC/PI positive cells under an inverted fluorescence microscope (**c**) or detected by Annexin V-FITC/PI double staining with flow cytometry (**d**). Mean ± SD (*n* = 3). Representative images were shown.*P < 0.05,**P < 0.01, ***P < 0.001, versus control group

Various CML cell lines, including IM-sensitive Bcr-Abl wild-type cell lines KBM5, BaF3-p210-WT, and K562, as well as IM-resistant Bcr-Abl-T315I cell lines KBM5R and BaF3-p210-T315I, were treated with various concentrations of NiPT for 48 h. A marked dose-dependent decrease in viability of all CML cell lines was observed in response to treatment with NiPT (Fig. 1a), with 50% inhibitory concentration (IC_50_) values of 0.14, 0.17, 0.3, 0.16, and 0.98 μM in KBM5, KBM5R, BaF3-p210-WT, BaF3-p210-T315I, and K562 cells, respectively.

The effect of NiPT on cell viability was further examined in CML cell lines by trypan blue exclusion staining assay. All CML cells were exposed to NiPT followed by trypan blue staining, a time- and dose-dependent inhibition of cell viability were observed (Fig. 1b), reflecting its anti-leukemia activity in CML cells.

### NiPT induces apoptosis in both Bcr-Abl-WT and Bcr-Abl-T315I cells

We next assessed the ability of NiPT to induce cell death in Bcr-Abl wild-type and T315I mutant cell lines. KBM5, KBM5R, K562, BaF3-p210-WT, and BaF3-p210-T315I cells were exposed to different doses of NiPT, a time-dependent increasing proportion of cell death was observed by recording the number of Annexin V/PI positive cells under an inverted fluorescence microscope. The 24-h time point images were shown in Fig. 1c. Similar results were obtained by flow cytometry analysis (Fig. 1d), supporting that NiPT induced apoptotic cell death.

### NiPT-induced apoptosis is associated with caspase activation and decreased expression of anti-apoptotic proteins in CML cells

**Fig. 2:**
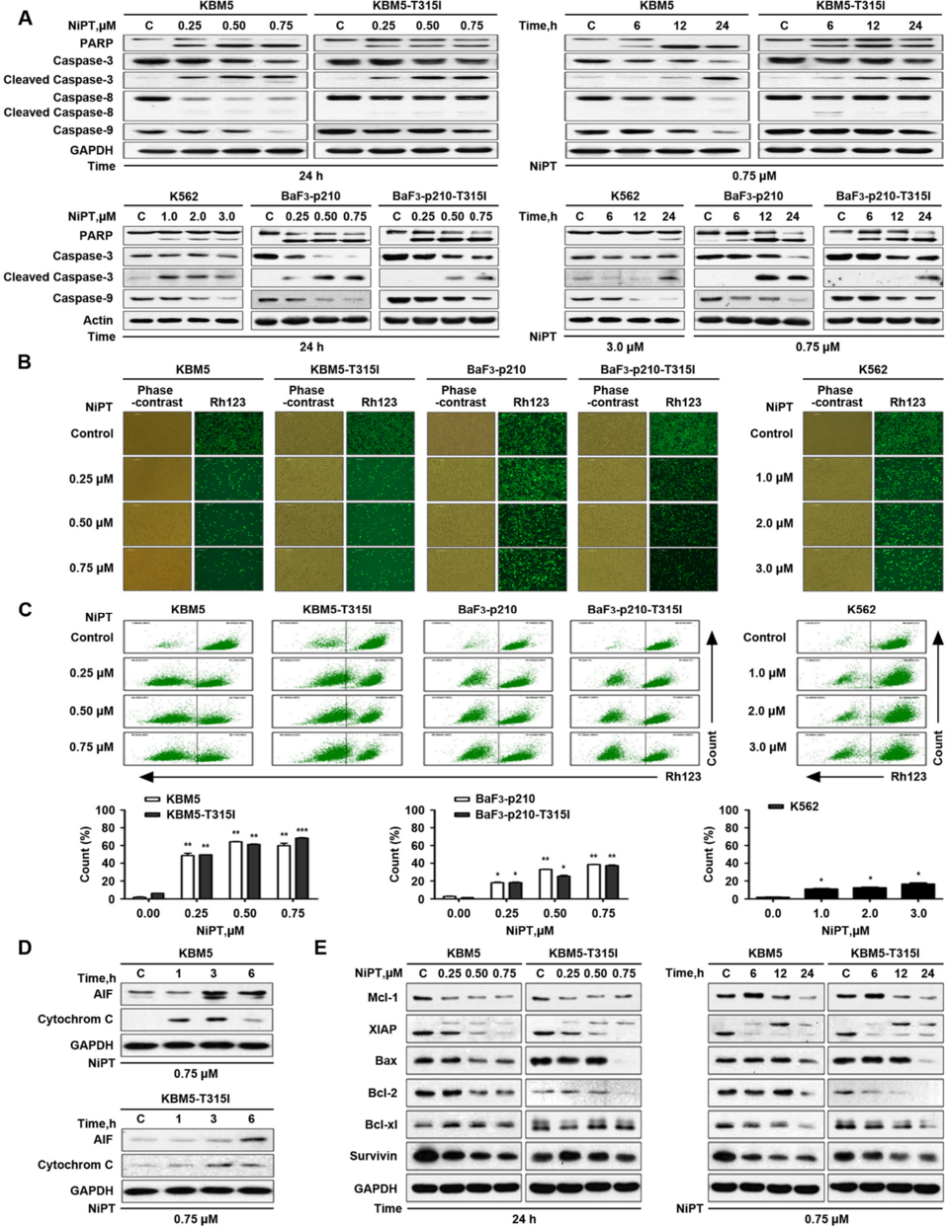
NiPT-induced apoptosis is associated with caspase activation. **a** NiPT leads to caspase-dependent cell apoptosis. KBM5, KBM5R, K562, BaF3-p210-WT, and BaF3-p210-T315I cells were exposed to NiPT at the indicated dose for the indicated duration; cell lysates were then analyzed with western blots for PARP, caspase-3, cleaved-caspase-3, caspase-8, and caspase-9. **b**, **c** NiPT reduces mitochondrial membrane potential in CML cells. Cells were treated with different doses of NiPT for 24 h, mitochondrial membrane potential was assessed by rhodamine-123 staining coupled with either fluorescence microscope (**b**) or flow cytometry (**c**). Mean ± SD (*n* = 3).*P < 0.05,**P < 0.01, ***P < 0.001, versus control group. **d** NiPT induces the release of cytochrome C and AIF in CML cells. KBM5 and KBM5R cells were treated with 0.5 μM NiPT for 1, 3, and 6 h, cytoplasmic proteins were analyzed by immunoblotting with cytochrome C and AIF antibodies. **e** NiPT downregulates anti-apoptotic proteins in CML cells. KBM5 and KBM5R cells were treated with the indicted doses of NiPT for the indicated duration; cell lysates were then immunoblotted to assess the changes of Mcl-1, XIAP, Bcl-2, Bcl-xl, and survivin

To understand the mechanism of action underlying NiPT-induced CML cell death, both Bcr-Abl wild-type and Bcr-Abl T315I cell lines were treated with various concentrations of NiPT for different durations, followed by measurement of apoptosis-associated proteins. NiPT markedly increased the cleavage of PARP, a hallmark of apoptosis. In accordance with these findings, our data showed that NiPT activates caspase 3, caspase 8, and caspase 9 in both dose- and time-dependent manner (Fig. 2a).

It is a widely accepted concept that mitochondrion is the regulating center of apoptosis. We next examined whether NiPT affects the integrity of mitochondrial membranes. Our result showed that the integrity of mitochondrial membranes was decreased in all CML cell lines after the treatment with NiPT (Fig. 2b, c). The release of cytochrome C and apoptosis induce factor (AIF) from mitochondria to the cytoplasm has been recognized as the early signs of apoptosis [34]. We therefore determined whether NiPT triggers the mitochondrial pathway. As displayed in Fig. 2d, cytochrome C and AIF release elevated at earlier time points, indicating that NiPT can activate the mitochondrial apoptosis pathway in CML cells.

To further investigate the mechanism of NiPT-induced apoptosis, the expression of other apoptosis-related proteins was detected. Results showed that there was a marked decline of anti-apoptotic proteins Mcl-1, XIAP, Bcl-2, and survivin (Fig. 2e).

### NiPT-induced proteasome inhibition is associated with ER-stress and cell apoptosis in CML cells

**Fig. 3:**
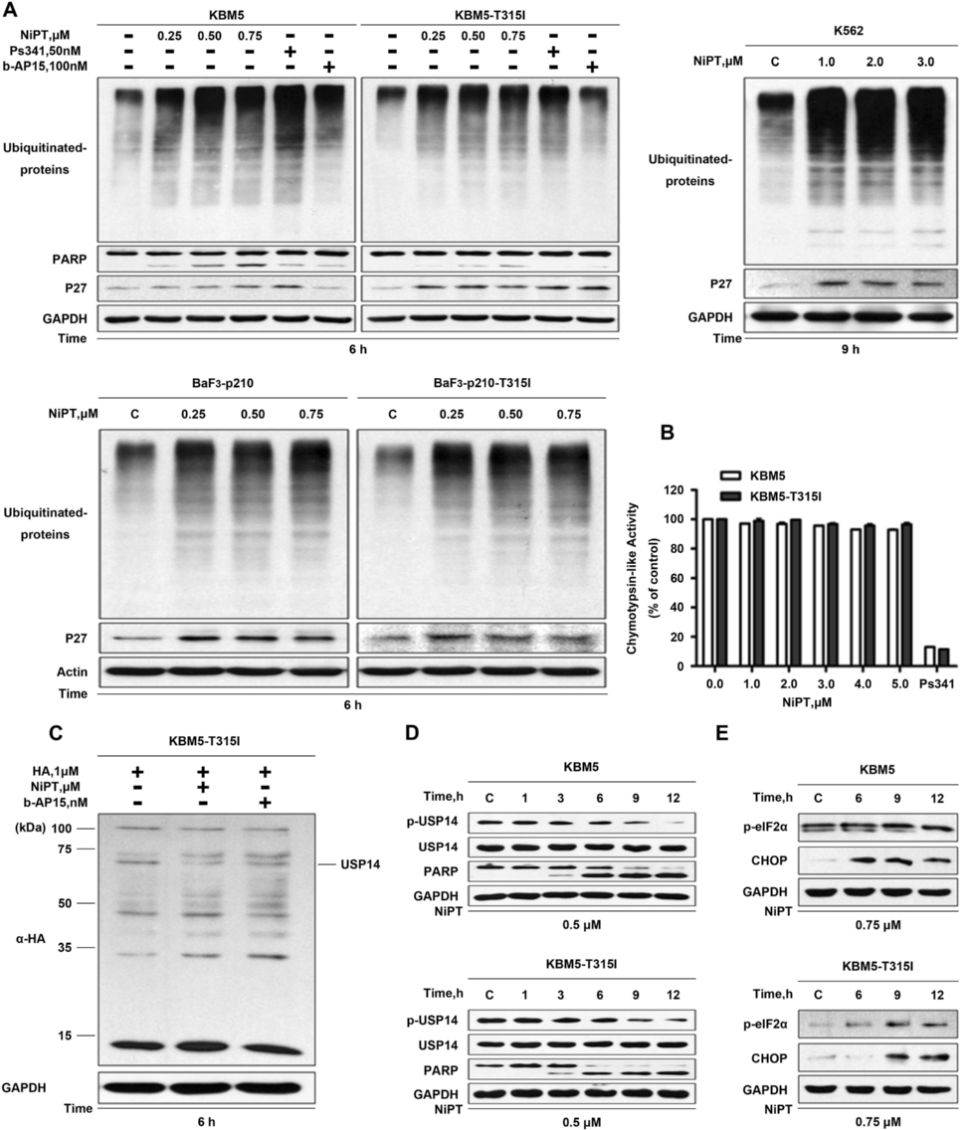
NiPT inhibits proteasome function in CML cells. **a** NiPT accumulates proteasome substrate proteins in CML cells. Cells were treated with various doses of NiPT for 6 h. The protein levels of PARP, ubiquitinated proteins (Ubs), and p27 were detected using western blots. Actin was used as a loading control. **b** NiPT has no obvious effect on the 20S proteasome peptidase activities in KBM5 and KBM5R cells. Cell lysate was treated with NiPT, and then the CT-like activity at different times was recorded using the fluorogenic Suc-LLVY-AMC substrate. Mean ± SD (*n* = 3). **c** Active-site-directed labeling of proteasomal DUBs. Cells were treated with NiPT, and then the cell lysate-contained DUBs were labeled with HA-UbVS. Labeled HA was detected via western blotting. b-AP15 (0.15 μM) was used as a positive control. **d** NiPT inhibits the phosphorylation of USP14. Cells were treated with the indicated dose of NiPT for the indicated duration. p-USP14 and total USP14 were detected with western blot. **e** NiPT treatment induces ER-stress response. KBM5 and KBM5R cells were treated with 0.75 μM NiPT for the indicated duration, then cell lysates were immunoblotted to assess the changes of p-eIF2α and CHOP

It is well established that inhibition of DUBs or the proteasome causes accumulation of ubiquitinated proteins [35]. Like in other cancer cells, we previously reported [27], we found that NiPT dose- and time-dependently induced accumulation of ubiquitinated proteins (Ubs) and proteasome substrate protein p27 in all CML cell lines we detected before the emergence of PARP cleavage (Fig. 3a). Specifically, NiPT treatment did not alter the proteasome peptidases in KBM5 and KBM5R cells either (Fig. 3b). The proteasome inhibitor bortezomib served as a positive control for chymotrypsin-like activity inhibition.

In accordance with our previous report that NiPT potently inhibits the ubiquitin-proteasome system via targeting the 19S proteasome-associated DUBs, NiPT treatment was able to compete with HA-UbVS for binding USP14 of DUB lysate from KBM5R cells (Fig. 3c). Furthermore, the phosphorylation of USP14 (S241) was substantially reduced in NiPT-treated CML cells, also indicative of suppression of the DUB activity of USP14 by NiPT treatment (Fig. 3d) [36].

Accumulation of ubiquitylated proteins induces unfolded protein response (UPR) and apoptosis [37]. Examination of the effect of NiPT on endoplasmic reticulum (ER) stress response showed that treatment of both KBM5 and KBM5R cells with NiPT activates PERK-mediated ER stress signaling, evidenced by significant induction of p-eIF2a (Fig. 3e).

Together, these results suggest that NiPT-mediated proteasome inhibition plays an important role in NiPT-induced ER stress response and caspase activation in CML cells.

### NiPT downregulates Bcr-Abl protein and inhibits its downstream signaling

**Fig. 4:**
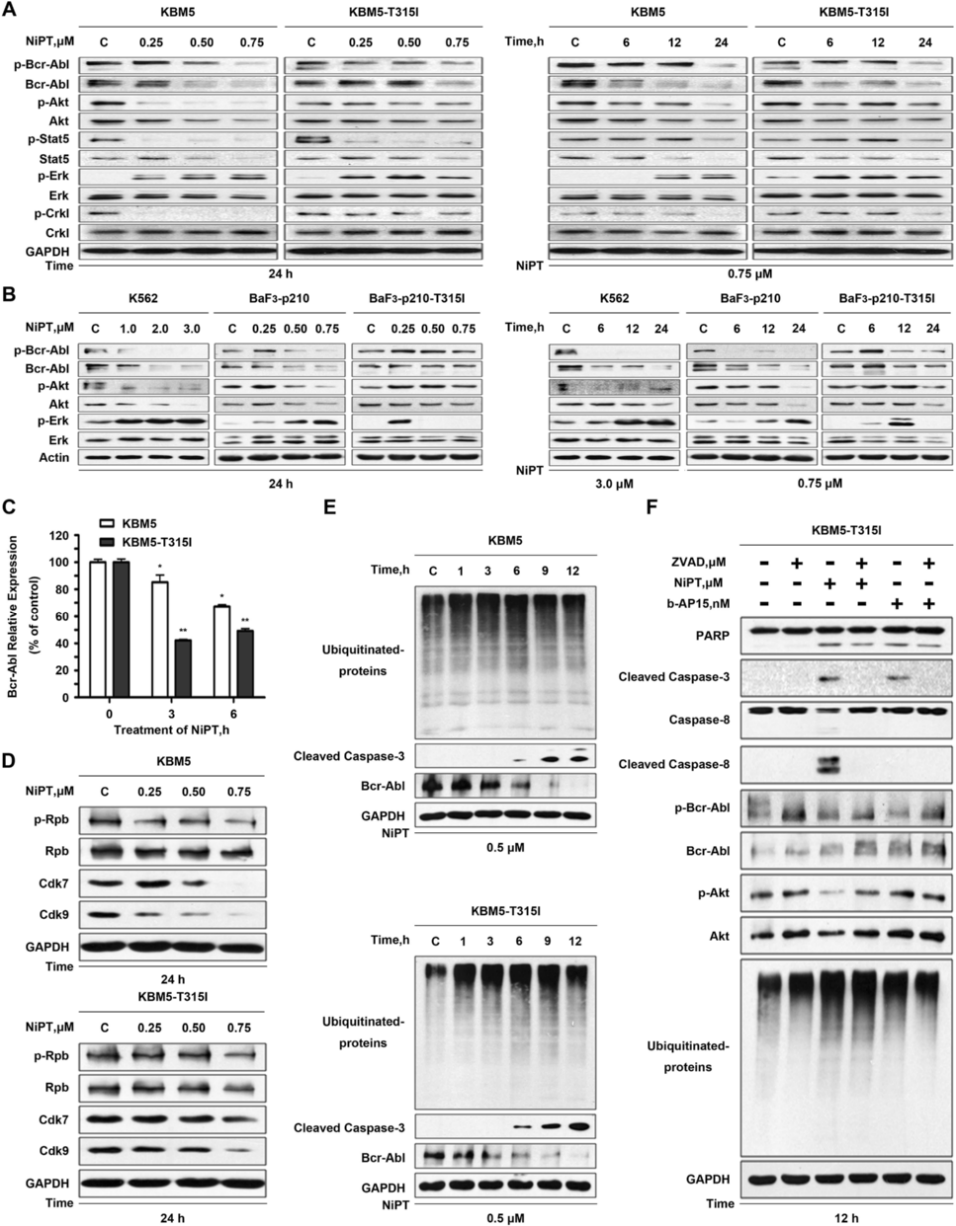
NiPT treatment downregulates Bcr-Abl and its downstream signaling proteins. **a**, **b** NiPT decreases the protein levels of Bcr-Abl and its downstream targets. CML cells were treated with NiPT as indicated, then harvested for western blot analyses for the indicated proteins. **c** NiPT decreases mRNA expression of Bcr-Abl. KBM5 and KBM5R cells were exposed to 0.5 μM NiPT for 3 or 6 h. The steady state Bcr-Abl mRNA level was measured using RT^2^-PCR, and its expression level relative to the control was calculated. Mean ± SD (*n* = 3). *P < 0.05,**P < 0.01, versus control group. **d** NiPT inhibits cellular activity of RNA pol II. KBM5 and KBM5R cells were dose-dependently treated with NiPT, the phosphorylated and total protein levels of RNA pol II, CDK7, and CDK9 were analyzed with western blot. **e** Proteasome inhibition mediated caspase activation induces Bcr-Abl downregulation. KBM5 and KBM5R cells were treated with 0.5 μM NiPT for the indicated durations. The Ubs, cleaved-caspase-3, and Bcr-Abl were analyzed with western blot. Representative images are shown. **f** NiPT decreases Bcr-Abl and the downstream signaling proteins in a caspase-dependent manner. KBM5R cells were treated with 0.5 μM NiPT for 12 h in the absence or presence of 25 μM pan-caspase inhibitor z-VAD-fmk. b-AP15 (150 nM) was used as a positive control of proteasomal DUB inhibition. The total and phosphorylated Bcr-Abl and its downstream proteins, Ubs, PARP, caspase-3, and caspase-8 were analyzed using western blots. Representative images are shown

We also found that NiPT downregulated the total and phosphorylation levels of Bcr-Abl protein in both Bcr-Abl-WT and Bcr-Abl-T315I cells in a dose- and time-dependent fashion (Fig. 4a, b). Further examination displayed that NiPT affected the expression of Bcr-Abl downstream target proteins. The phosphorylation of STAT5, Akt, and Crkl was also significantly decreased in a dose- and time-dependent manner, occurring earlier than the downregulation of their total forms. Interestingly, NiPT treatment also stimulated ERK phosphorylation, which may have something to do with the inhibition of the catalytic process of autophagy in leukemia cells, similar to the function of bortezomib reported previously [38].

### Bcr-Abl downregulation results from diminished gene expression and caspase-dependent cleavage

To address the mechanism underlying the NiPT-mediated Bcr-Abl protein downregulation in CML cells, we analyzed the expression of Bcr-Abl at the transcriptional level. KBM5 and KBM5R cells were treated with increasing concentrations of NiPT for 6 h. RT-PCR revealed that the mRNA level of Bcr-Abl was decreased to some extent in both cells (Fig. 4c). The degree of mRNA reduction is apparently less than the reduction of the corresponding protein levels but the downregulation of mRNA by NiPT likely contributes to the decrease of the Bcr-Abl proteins. To further address this issue, RNA polymerase II (RNA pol II) was analyzed. As shown in Fig. 4d, the phosphorylation of RNA pol II was diminished to a certain extent in both KBM5 and KBM5R cells by NiPT treatment. Together, NiPT leads to inhibition of RNA polymerase followed by downregulation of Bcr-Abl mRNA and protein levels, regardless of the mutation status of the Bcr-Abl gene.

We and others have reported that Bcr-Abl could be cleaved by caspase activation [29, 30]. To gain insight into the mechanism of NiPT-induced Bcr-Abl decline, we studied its dependence on the caspase activation. Specifically, we observed that pan-caspase inhibitor z-VAD-fmk mostly recovered NiPT-mediated cell death, decreases of Bcr-Abl as well as its downstream proteins to a certain extent but not ubiquitinated protein accumulation (Fig. 4e, f). These results demonstrate that caspase activation triggered by NiPT-induced proteasome inhibition is required for the downregulation of Bcr-Abl and its downstream events.

### Ex vivo effect of NiPT on primary monocytes from patients with CML

**Fig. 5:**
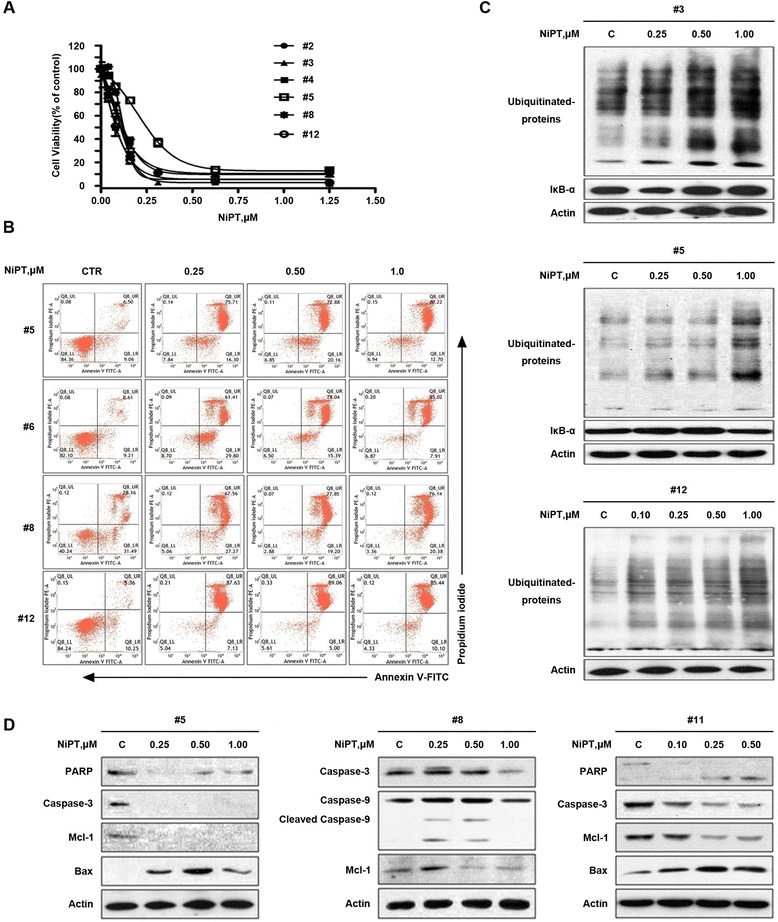
NiPT triggers cell apoptosis and proteasome inhibition in cancer cells from CML patients. **a** NiPT decreases cell viability of CML patient cancer cells. CML cells from 12 CML patients were treated with NiPT at the indicated doses for 48 h, and cell viability was detected by the MTS assay. Mean ± SD (*n* = 3). **b** NiPT induces cell apoptosis in CML patient cancer cells. Cancer cells from CML patients were isolated and incubated with NiPT for 36 h, followed by detecting the Annexin V/PI positive cells by flow cytometry. Representative images are shown. **c** NiPT accumulates ubiquitinated proteins and IκB-α in CML cancer cells. Cancer cells from three CML patients were treated with NiPT for 6 h. The indicated proteins were analyzed using western blots. (#3, *5*: imatinib-sensitive patients; #12: imatinib-resistant patient). **d** NiPT induces cleavage of PARP and caspase-3 or caspase-9, and downregulates Mcl-1 protein in patients’ cancer cells. Cells from CML patients were treated with NiPT, and the indicated proteins were detected with western blots (#5, 8: imatinib-sensitive patients; #11: imatinib-resistant patient)

The above results clearly indicate that NiPT-mediated proteasome inhibition and cytotoxicity is effective in both IM-sensitive and IM-resistant CML cells. We next evaluated the ex vivo anti-neoplastic effect of NiPT on bone marrow mononuclear cells from 12 patients with CML (4 patients are IM resistant). As displayed in Fig. 5a, NiPT decreased the cell viability of primary monocytes from CML patients with IC_50_ values ranging from 0.58 to 0.82 μM, while for the peripheral mononuclear cells from three healthy volunteers the IC_50_ values were more than 3.0 μM as we reported recently [27].

It was further found that NiPT treatment at doses from 0.25 to 1.0 μM for 36 h resulted in significant apoptosis in all the monocytes from the 12 CML patients as detected by Annexin V/PI double staining (Fig. 5b). NiPT treatment also significantly induced the accumulation of ubiquitinated proteins and proteasome substrate protein IκB-α (Fig. 5c), reduced the levels of pro-caspase-3 and pro-caspase-9, Mcl-1, and induced PARP cleavage in the primary monocytes (Fig. 5c, d). These results are consistent with the in vitro inhibitory effect of NiPT on both Bcr-Abl-WT and Bcr-Abl-T315I cell lines, suggesting the potential use of NiPT for treatment of CML patients.

### NiPT inhibits the growth of Bcr-Abl-WT and Bcr-Abl-T315I mutant xenografts in nude mice

**Fig. 6:**
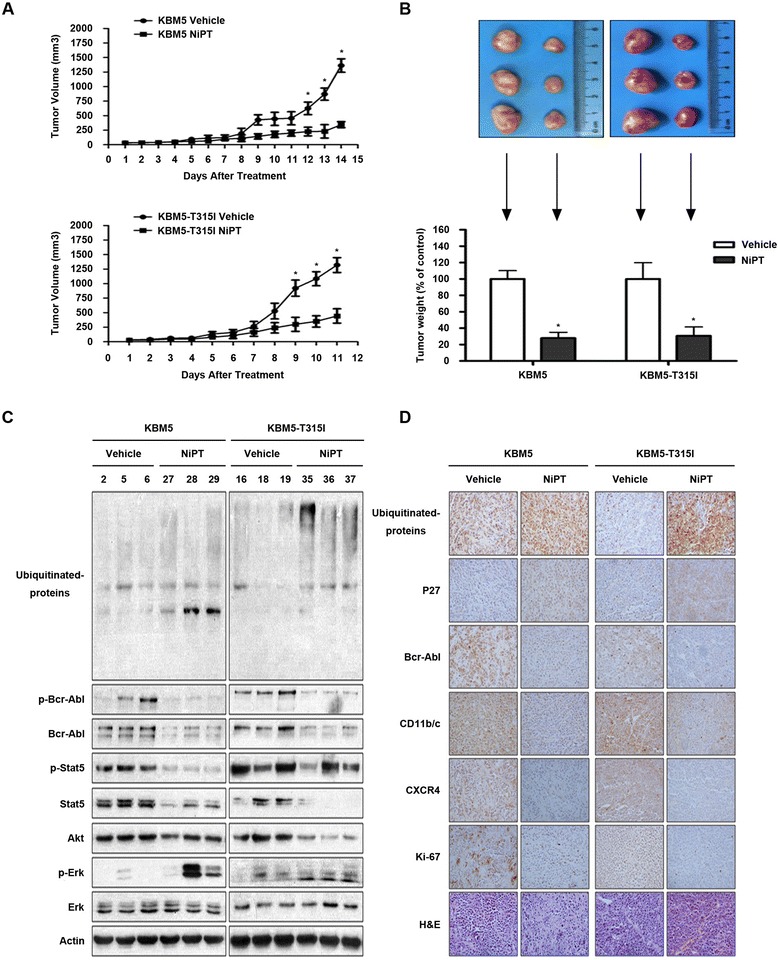
In vivo effect of NiPT on KBM5- and KBM5R cell-derived mouse xenograft models. Nude mice bearing wild type and T315I-mutant Bcr-Abl xenograft tumors were treated with either vehicle or NiPT (2.5 mg/kg/day) for 11–14 days after inoculation of KBM5 and KBM5R cells. **a** NiPT inhibits tumor growth in vivo. Tumor growth curves were recorded every day in two sets of experiments. Mean ± SD (*n* = 6). *P < 0.05, **P < 0.01, ***P < 0.001, versus NiPT-treated group. **b** On day 14 after inoculation, the mice were sacrificed, and the tumor tissues were weighed and imaged. ***P < 0.001, versus control group. **c**, **d** Western blot analyses (**c**) and/or immunohistochemistry staining for proteasome substrates (ubiquitinated proteins, p27), Bcr-Abl-related proteins (Bcr-Abl, p-Bcr-Abl) and its downstream proteins (Stat5, p-Stat5, Akt, Erk, p-Erk), and protein biomarkers related to proliferation (Ki-67), differentiation (CD11b/c), and adhesion and migration (CXCR4) in the xenograft tumor tissues from the KBM5 vehicle group (#2, 5, 6), KBM5 NiPT-treated group (#27, 28, 29), KBM5-T315I vehicle group (#16, 18, 19), and KBM5R-T315I NiPT-treated group (#35, 36, 37). In panel **d**, the proteins of interest are *immunostained brown*. All the immunohistochemical stainings were repeated in three mouse tumor tissues and the representative images are shown

We next evaluated the effect of NiPT in vivo using a nude mouse xenograft model. KBM5 and KMB5R cells were inoculated subcutaneously in nude mice. A marked reduction in tumor growth was noted in NiPT-treated mice versus mice receiving vehicle alone (Fig. 6a, b) while body weight remained relatively stable in each group (data not shown). Protein levels of Bcr-Abl and its downstream targets Akt and STAT5 were significantly decreased in the NiPT-treated tumors (Fig. 6c, d) while the phosphorylation of ERK was highly accumulated, consistent with the in vitro effect of NiPT. The ubiquitinated proteins and proteasome substrates p27 were highly accumulated in NiPT-treated tumor tissues versus the control-treated models (Fig. 6d), indicating that NiPT inhibits proteasome function in both IM-sensitive and IM-resistant xenografts. The protein biomarkers related to proliferation, differentiation, and adhesion, such as Ki67, CD11b/c, and CXCR4 were also downregulated by the NiPT treatment in both KBM5 and KBM5R models (Fig. 6d). Together, these results demonstrate that NiPT inhibits xenografted Bcr-Abl-WT and Bcr-Abl-T315I harboring cells in vivo.

## Discussion

The development of tyrosine kinase inhibitors (TKIs) has led to extended lifespans for many patients with CML. Resistance against TKIs represents a relevant clinical problem in treatment of CML. On the basis of their activity against the spectrum of Bcr-Abl mutations which have shown to be the most prominent mechanism of resistance to imatinib [13–15], new TKIs have been classified as second generation (such as nilotinib, dasatinib, and bosutinib) or third generation (such as ponatinib) TKIs. Although the second-generation TKIs can effectively inhibit the phosphorylation of most of the mutated Bcr-Abl (e.g., E255K, M351T), they had a little effect on Bcr-Abl-T315I [18–20]. The third-generation TKIs ponatinib has activity against all of the known Bcr-Abl mutations including T315I. However, the resistance to ponatinib would limit the application of the agent to a certain extent [21, 22]. In addition, Bcr-Abl-expressing cells are relatively resistant to apoptosis induced by conventional cytotoxic agents [39, 40]. These observations suggest that additional approaches and targeting strategies are needed to provide more treatment options for CML patients. In light of the possibility to overcome kinase inhibitor resistance, several compounds including heat shock protein 90 (Hsp90) inhibitors, arsenic trioxide, proteasome inhibitors. and others have been found to lead to CML cell death through downregulation of Bcr-Abl expression or a loss of Bcr-Abl stability other than direct kinase inhibition [41]. Taken together, Bcr-Abl downregulation is the common effect and is likely the major factor inducing apoptosis in Bcr-Abl-expressing cells. Additional compounds that induce rapid changes in Bcr-Abl levels with limited impact on other proteins are still needed.

Previously, we have reported that NiPT could produce tumor tissue-specific proteasome-associated DUBs inhibition and tumor-specific toxicity, which might have clinical significance for designing novel strategies for cancer treatment [27]. In this study, we analyzed the mechanism of action of NiPT, the nickel ion, and PT-chelating product with previously reported inhibitory activity of 19S proteasome-associated DUBs, in overcoming IM-resistant CML cells in vitro, ex vivo and in vivo. In the in vitro study, NiPT dose- and time-dependently decreased cell viability and induced cell death in both IM-sensitive and IM-resistant cell lines; in the mononuclear cancer cells from either IM-sensitive or IM-resistant patients with CML, NiPT also had the same effects as in cultured cell lines; in the in vivo experiment, both IM-sensitive and IM-resistant xenografted tumors were all sensitive to NiPT treatment. These results have clearly demonstrated that NiPT can efficiently induce cytotoxicity in IM-resistant cancer cells. To our knowledge, this is the first report to show that NiPT, as a novel inhibitor of 19S proteasome-associated DUBs, is effective in vitro and in vivo against CML cells, including those with the Bcr-Abl-T315I mutation.

UPS inhibitors have been reported to be effective in overcoming IM-resistant cancer cells *via* either inhibiting Bcr-Abl expression or interfering other mechanisms [28, 29, 32], but the mechanism is far from being fully understood. Recently, we have reported that NiPT is able to inhibit the activity of 19S proteasome-associated DUBs USP14 and UCHL5 but not the 20S proteasome [27]. We confirmed that NiPT induces cell apoptosis and overcomes IM-resistance in CML cells through both Bcr-Abl-dependent and Bcr-Abl-independent mechanisms. On the one hand, NiPT inhibits the transcription of the Bcr-Abl gene, and future studies need to investigate the possibility that NiPT activates caspases which cleaves RNA pol II leading to decrease of Bcr-Abl mRNA. On the other, NiPT-induced caspase activation cleaves Bcr-Abl (Fig. 4c–f), thus leading to Bcr-Abl downregulation and cell proliferation inhibition. Here, we proposed a novel pathway that NiPT-mediated UPS inhibition and caspase activation are responsible for the downregulation of Bcr-Abl protein, which contributes to overcoming IM-resistance by NiPT. Like in other cancer cells, NiPT induced UPS inhibition in both Bcr-Abl wild-type and T315I cell lines, as well as in primary mononuclear cancer cells derived from CML patients including IM-resistant or IM-sensitive in vitro. It was further confirmed that NiPT also inhibited USP function in the xenografted tumor model bearing wild-type- and T315I-Bcr-Abl genes in vivo. We have reported that proteasome inhibition induced ER-stress response plays an important role in caspase activation and cell apoptosis [42]. The induced ER-stress response accumulates Ca^2+^ in cytoplasm to alter mitochondrial membrane permeability, leading to the release of cytochrome C and AIF, followed by caspase activation and apoptosis. Here, we also found that NiPT time-dependently induced the ER-stress response pathway, while the release of cytochrome C and AIF was induced and the anti-apoptotic proteins like Bcl-2, XIAP, and Mcl-1 were dramatically decreased. The released apoptotic factors bind with caspase-9 forming a protein complex, inducing caspase activation (Fig. 2). After caspase activation, on the one hand, it induced PARP cleavage and apoptosis; on the other hand, it directly cleaved Bcr-Abl protein which has been confirmed in our work (Fig. 4e, f).

Bcr-Abl is a constitutively active tyrosine kinase that phosphorylates several substrates, and activates multiple signal transduction pathways such as MAPK/ERK, PI3K/AKT, and STAT3/STAT5, all of which are responsible for cell proliferation and survival [4–6]. The downstream target of STAT5 responsible for enhanced survival of Bcr-Abl cells are involved in the transcription of Mcl-1, survivin, or Bcl-2 [7–9]. Treatment with NiPT resulted in downregulation of Mcl-1, Bcl-2, and XIAP (Fig. 2e). Even though NiPT may impact on multiple molecules, in the case of Bcr-Abl, loss of signaling is associated with the onset of CML cell apoptosis. Research suggests that sustained activation of ERK inhibits the maturation step of the autophagy process [38]. Interestingly, we have also noted that NiPT, like bortezomib, triggers ERK phosphorylation distinctly, but this is not sufficient to fully protect CML cells from DUB inhibition-mediated apoptosis. NiPT treatment stimulated ERK phosphorylation may attribute to the inhibition of the catalytic process of autophagy in leukemia cells, just like the action of bortezomib reported previously [43]. In this regard, the inhibition of autophagy by NiPT may enhance the chemotherapy efficacy of this agent in CML cells. Future studies need to be performed to investigate the mechanism involved in autophagy inhibition by NiPT. Taken together, both caspase-induced apoptosis and Bcr-Abl downregulation likely contribute to the cytotoxic effects of NiPT on IM-sensitive or IM-resistant cancer cells.

At present, most studies on Bcr-Abl inhibition are centered in finding tyrosine kinase inhibitors that directly inhibit tyrosine kinase activity. Here, we propose an alternative strategy to enhance Bcr-Abl cleavage by activating the caspase system. In summary, here, we have demonstrated the mechanisms of overcoming IM-resistance by NiPT both in the Bcr-Abl-T315I cells and in the xenografted Bcr-Abl-T315I harboring cells. These findings suggest for the first time that NiPT may have clinical benefit for patients with CML, particularly in those suffering from imatinib-resistance and the recurrent forms of this disease, providing great importance in the future clinical CML therapy.

Notably, we previously reported the anti-tumor effects of NiPT in other types of tumor models, such as lung cancer (A549), hepatocarcinoma (SMMC-7721), and myeloma (U266) [27]. We found that although higher doses were needed for those cancers compared with CML cells, NiPT could inhibit cell growth of all these cancer models, which raises the possibility that NiPT could have wide application for the treatment of different kinds of cancer in the future.

## Conclusions

NiPT induces Bcr-Abl decrease in Bcr-Abl wild-type and Bcr-Abl-T315I mutation cells through downregulation of Bcr-Abl transcription and Bcr-Abl protein cleavage mediated by proteasome inhibition-induced caspase activation. Meanwhile, 19S proteasome-associated DUB inhibition plays an important role in NiPT-induced caspase activation and apoptosis. We here propose an alternative strategy to overcome IM resistance by enhancing Bcr-Abl downregulation, which should have great clinical significance in IM-resistant cancer therapy.

## Abbreviations

AIF: Apoptosis induce factor
CML: Chronic myelogenous leukemia
DMSO: Dimethyl sulfoxide
DUB: Deubiquitinase
ER: Endoplasmic reticulum
GA: Gambogic acid
HA-Ub-VS: HA-ubiquitin-vinyl sulfone
Hsp90: Heat shock protein 90
IM: Imatinib
KBM5R: KBM5-T315I
NiPT: Nickel pyrithione
PI: Propidium iodide
PS-341: Bortezomib
PT: Pyrithione
RNA pol II: RNA polymerase II
RT^2^-PCR: Reverse transcriptase real-time DNA polymerase chain reaction
TKIs: Tyrosine kinase inhibitors
Ubs: Ubiquitinated proteins
UCHL5: Ubiquitin carboxyl-terminal hydrolase isozyme L5
UPR: Unfolded protein response
UPS: Ubiquitin proteasome system
USP14: Ubiquitin specific peptidase 14
ZnPT: The zinc complex of pyrithione
